# Pan-neurofascinopathies: a subgroup that mimic other neuroimmune-mediated diseases

**DOI:** 10.1055/s-0046-1829127

**Published:** 2026-10-23

**Authors:** Marcus Vinícius Magno Gonçalves, Pedro José Tomaselli, Trajano Aguiar Pires Gonçalves, Rodrigo Siqueira Soares Frezatti, Wilson Marques Junior

**Affiliations:** 1 Universidade de São Paulo, Faculdade de Medicina, Programa de NeurociênciasRibeirão Preto SPBrazil; 2 Universidade de São Paulo, Faculdade de Medicina, Departamento de NeurociênciasRibeirão Preto SPBrazil; 3 Universidade de São Paulo, Instituto Nacional de Ciência e Tecnologia Translacional em MedicinaRibeirão Preto SPBrazil

**Keywords:** Neuroimmunology, Autoimmune Diseases, Autoimmune Diseases of the Nervous System

## Abstract

Pan-neurofascinopathies represent a distinct subset of inflammatory neuropathies characterized by autoantibodies that target multiple neurofascin (NF) isoforms, which are essential for nodal and paranodal architecture in peripheral nerves. These antibodies are associated with a broad clinical spectrum, often mimicking Guillain-Barré syndrome (GBS) or chronic inflammatory demyelinating polyradiculoneuropathy (CIDP), and exhibit significant variability depending on the immunoglobulin G (IgG) subclass involved. The IgG1 and 3 subclasses correlate with acute, fulminant presentations and complement activation, whereas IgG4-mediated cases are typically indolent but resistant to first-line therapies. Although primarily restricted to the peripheral nervous system, pan-neurofascinopathies may also mimic central demyelinating diseases and present with systemic features such as nephrotic syndrome. These atypical manifestations highlight the expanding clinical spectrum of the disease. Early identification of anti-pan-NF antibodies and their respective IgG subclasses is crucial to guide diagnosis and management. Timely recognition enables implementation of aggressive B cell depletion-based treatment strategies, which are essential to avoid long-term neurological sequelae in affected patients.

## INTRODUCTION

Inflammatory neuropathies represent a heterogeneous group of diseases that share immune-mediated dysfunction of the peripheral nerve due to the loss of tolerance to antigens directly involved in nerve structure.

The structural compartmentalization of inflammatory neuropathies began in 2013, when Uncini et al. characterized neuropathies associated with antiganglioside antibodies, identifying distinct findings of nodal dysfunction and describing the combination of impaired saltatory conduction with axonal degeneration, thus proposing the concept of nodopathy/paranodopathy. 1 This classification was later expanded to include antigens present in adhesion molecules associated with the peripheral nerve. 2

Neurofascins (NFs) are a group of nodal/paranodal adhesion molecules that play a crucial role in maintaining myelin sheath stability and the organization of ion channels in the peripheral nervous system. 3 These molecules contain antigens which, when recognized, are associated with a broad clinical and neurophysiological spectrum of inflammatory peripheral neuropathies. 2 Variations in clinical presentation, prognosis, and treatment response partially depend on the specific NF subtype involved (e.g., NF140, NF155, NF166, NF180, and NF186). However, in many cases, due to shared antigenic sequences, concomitant involvement of multiple NF subtypes may occur, leading to more severe and rapidly progressive conditions, collectively referred to as anti-pan-NF neuropathy. 4 The clinical variability is influenced not only by the combination of NF subtypes affected but also by the immunoglobulin G (IgG 1–4) subclass mediating the inflammatory response. 4 5

In this review, we describe the clinical and neurophysiological features of anti-pan-NF neuropathies and examine the role of antibody subclasses, along with their therapeutic implications.

## MICROANATOMY OF THE NODE/PARANODE AND ITS RELATIONSHIP WITH NEUROFASCINS

Myelinated nerve fibers are organized into the node, paranode, juxtaparanode, and internode regions. In the nodal region, the axolemma is in direct contact with the extracellular fluid. In the paranodal region, non-compacted myelin loops are attached to the axolemma, initiating a compaction process in the juxtaparanodal region, which becomes fully compacted in the internode ( Figure 1A ). 2 This anatomical organization allows for an asymmetric distribution of ion channels, enabling saltatory conduction. The nodal region is densely populated with voltage-gated sodium channels, with a low density of potassium channels. 2 In the juxtaparanodal regions, this distribution is reversed. 2 The internode also contains sodium and potassium channels, but at low density, which is not sufficient to support action potential propagation. To make this microarchitecture functional, axoglial protein complexes composed of NF, contactin, Caspr1, and gangliosides delineate the functional regions and anchor the ion channels. 2

**Figure 1:**
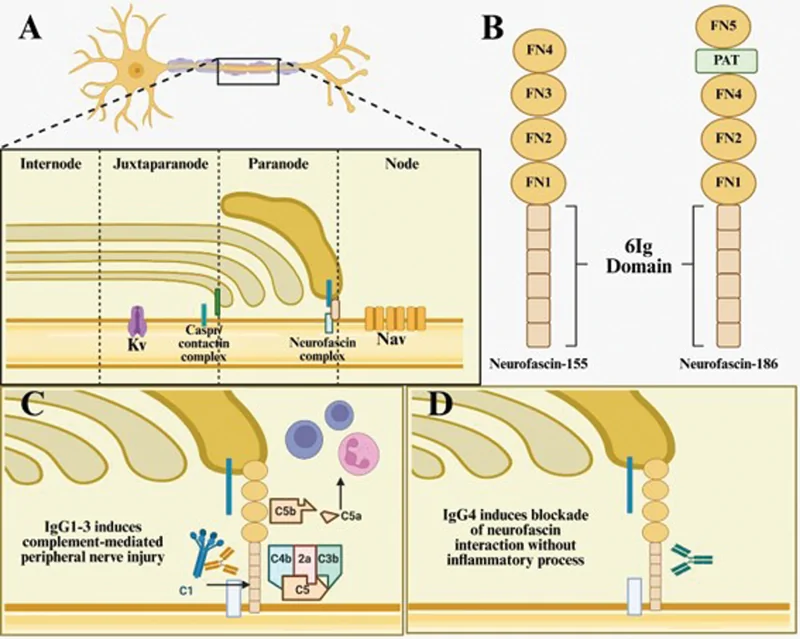
Microanatomy of the myelinated peripheral nerve and its relationship with paranodal adhesion molecules. ( **A** ) The microanatomy of the myelinated peripheral nerve comprises four main regions: node, paranode, juxtaparanode, and internode. It is in the paranodal region that neurofascin adhesion molecules are located, allowing for the proper compartmentalization of voltage-gated sodium channels (Nav) at the nodal region. This, together with the compartmentalization of voltage-gated potassium channels (Kv) at the juxtaparanodal region, enables efficient saltatory conduction. ( **B** ) Neurofascins (NFs) are cell adhesion glycoproteins composed of six immunoglobulin-like domains (6Ig), which are common to all subtypes, and a variable fibronectin type III (FN) domain, which characterizes the different neurofascin isoforms. The 6Ig domain is typically the target recognized by antipanneurofascin antibodies. ( **C** ) In panneurofascinopathies mediated by IgG1 to 3, activation of the classical complement pathway (initiated by C1q) occurs, followed by the formation of the membrane attack complex (MAC) and recruitment of inflammatory cells. ( **D** ) In patients with IgG4-mediated panneurofascinopathies, complement activation and inflammatory cell recruitment do not occur. In these cases, the pathogenic mechanism is thought to involve disruption of myelin anchorage at the paranodal region due to interference with neurofascin-mediated adhesion.

As for NF, they are adhesion molecules belonging to the L1 group of the immunoglobulin superfamily, with several possible isoforms. These isoforms share the same transmembrane and immunoglobulin-like domains but differ in their fibronectin domain **s** 6 ( Figure 1B ). This variation results in six isoforms (NF140, NF155, NF166, NF180, and NF186), each playing distinct roles throughout the maturation and development of the peripheral nerve. The isoforms NF140, NF166, and NF180 are expressed during embryonic development, whereas NF155 and NF186 are involved in the structure and function of the mature node/paranode regions. 6

Furthermore, NF155 is located in the paranodal region, where it forms a protein complex with contactin and Caspr1, acting as a structural bridge that anchors the noncompacted myelin loops to the axolemma. 7 As for NF186, it is primarily located in the nodal region, where it forms protein complexes with axonal cytoskeletal proteins and components of the extracellular matrix. 7 Additionally, it plays a critical role in the positioning of voltage-gated sodium channels. The nodal/paranodal distribution of these NF isoforms plays a fundamental role in the compartmentalization of the peripheral nerve and, consequently, in the proper saltatory conduction of action potentials. 7

## IMMUNOGLOBULIN G (IgG) SUBTYPES AND THEIR INFLUENCE ON PHENOTYPE AND THERAPEUTIC RESPONSE

Immunoglobulin G (IgG) is one of the most abundant proteins in plasma, accounting for around 10 to 20% of the total plasma protein content. 5 Structurally, it consists of two heavy chains and two light chains joined by disulfide bonds. The heavy chains are interrupted by a hinge region, which allows the immunoglobulin to adopt a characteristic “Y” shape. The distal portions of the “arms” of the IgG molecule contain variable regions from both the heavy and light chains, whereas the proximal portions contain constant regions ( Figure 2A ). 5

**Figure 2:**
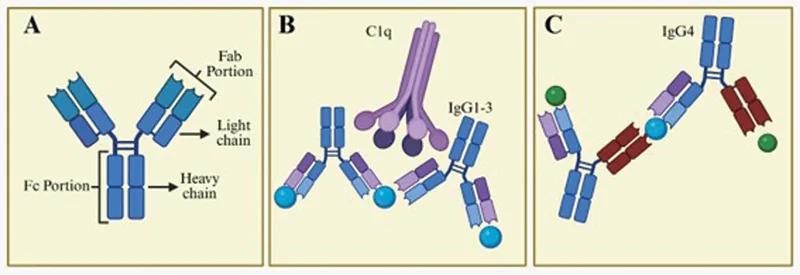
The structure of immunoglobulin G (IgG) and characteristics of each subclass. ( **A** ) The molecules are composed of two heavy chains and two light chains. The Fab region is variable and responsible for antigen binding, while the Fc region is constant and mediates interactions with components of the innate immune system, such as macrophages and complement proteins. ( **B** ) IgG1 to 3 subclasses are characterized by their ability to activate the complement system. Additionally, IgG1 to 3 molecules are bivalent and monospecific, meaning they can bind two identical epitopes of the same antigen. This feature facilitates antigen crosslinking, promoting the formation of immune complexes and enhancing the inflammatory response. ( **C** ) IgG4 is functionally monovalent and bispecific, as it is unable to crosslink identical antigens due to Fab-arm exchange. Furthermore, IgG4 is inefficient in complement activation, contributing to its noninflammatory nature.

This protein can be classified into four subclasses (IgG 1–4), ordered by decreasing abundance. These subclasses differ not only in microstructural aspects—such as molecular weight, number of disulfide bonds, and amino acid length in the hinge region—but also in their effector functions. 5 The route of antigen presentation and the antigen's molecular composition are key factors in determining which subclass will be stimulated. 5 The IgG1 to 3 subclasses are capable of strongly activating the complement system, unlike IgG4. Furthermore, IgG3 is considered the most potent effector subclass, associated with intense inflammatory responses, although its action is limited by a short half-life. 5 As for IgG4, it is the least abundant subclass and, due to its unique structural characteristics, is associated with anti-inflammatory responses. 5

Because the heavy and light chains of IgG4 are joined by noncovalent bonds, the Fab region can bind to antigens using only one arm (monovalent), in contrast to IgG1 to 3, which are bivalent and capable of simultaneously binding multiple antigens ( Figure 2 B,C ). 5 8 This monovalency leads to a lower local antigen concentration, which—combined with IgG4's limited capacity to mediate cell-dependent cytotoxicity and activate complement—does not elicit robust inflammatory responses. Its pathogenic mechanism primarily involves the disruption of protein–protein interactions through interference with antigen binding. 5 8

Patients with antipan-NF neuropathies harboring IgG3 antibodies typically exhibit a more aggressive and rapidly progressive clinical course, with the most pronounced progression observed in the IgG3 subtype. Its phenotypes are associated with more severe clinical presentations, with a high prevalence of ventilatory failure (modified Egris scale > 3), initial presentation with a high modified Hughes scale score above 3, older patients with cranial nerve involvement in 100% of patients, and a likely high prevalence of mortality (> 25%). 4

Although the massive activation of complement followed by inflammatory cell recruitment can lead to dramatic Guillain-Barré-like presentations, these IgG1 to 3 subclasses are also associated with better responses to plasmapheresis than intravenous immunoglobulin (IVIG), which is why a future Guillain-Barré syndrome (GBS) consensus would plausibly recommend plasmapheresis as first-line treatment, not only in terms of timing (2–4 weeks from symptom onset) but also based on clinical scores with higher scores (modified Hughes and Egris scales > 3) with the aim of reducing significant early axonal loss due to therapeutic vacancy. This is because catastrophic clinical presentations may have a lower response to IVIG, which currently remains a first-line therapy regardless of the clinical score. 4

In contrast, antipan-NF IgG4 neuropathies cause peripheral nerve dysfunction through the disruption of NF interactions with other adhesion molecules, leading to detachment of the paranodal myelin without evidence of inflammation or complement activation. 8 Despite a more indolent clinical course, IgG4-associated antipan-NF neuropathies are typically resistant to conventional first-line therapies for inflammatory neuropathies, with B cell–depleting therapies showing greater efficacy. 4 8

The relationship between IgG subclass, clinical phenotype, and treatment response is complex, as subclass switching may occur over the course of the disease. 4 5 It is not uncommon for patients to initially present with fulminant symptoms driven by IgG3, which are responsive to IVIG, but later transition to an IgG4-dominant profile, resulting in treatment resistance and a slowly progressive clinical course. 4 5

## CLINICAL AND LABORATORIAL ASPECTS ASSOCIATED WITH ANTI-PAN-NEUROFASCINOPATHIES

Pan-NF antibodies are rare, and their true prevalence remains undefined. Currently, due to the gold standard testing technique being cell-based assay (CBA), these autoantibodies are expensive, making the availability of biochips a remote possibility, likely because it is a continental developing country with universal public health coverage. The difficulty in accessing autoantibodies may contribute to underdiagnosis in countries like Brazil.

The classical clinical presentation associated with pan-NF antibodies involves acute forms of inflammatory neuropathy, often severe and with potential for recurrence, which may lead to misdiagnosis of GBS and its variants in monophasic presentations and acute and subacute onset, as well as in cases of onset lasting more than 4 to 6 weeks and with recurrence, typical chronic inflammatory demyelinating polyradiculoneuropathy (CIDP) and its variants, or a misdiagnosis of CIDP-A. 4

Furthermore, pan-NF phenotypes, by mimicking both GBS (in monophasic forms) and CIDP (chronic relapsing and progressive forms), can unfortunately have catastrophic clinical outcomes with early axonal loss, permanent motor disability and even death if not treated with plasmapheresis or early anti-CD20 therapies, which is not currently contemplated in the latest GBS and CIDP consensus guidelines. Involvement outside the peripheral nervous system has also been described (such as proteinuria and nephrotic syndrome) and may serve as an additional clue for diagnosis. 4

They can occur at any age, though the vast majority of reported cases involve patients over 60-years-old. Males are more frequently affected than females, with higher prevalence of respiratory failure (high Egris scale), cranial nerve involvement (100%), and finally (high Hughes scale): initial non-ambulatory presentation without support, wheelchair users, or even severely tetraplegic and quadriplegic patients including phenotypes with locked in. Unfortunately, such catastrophic clinical presentations present with a high mortality rate (> 25%). 4 To date, the true prevalence of pan-neurofascin antibody–associated phenotypes that mimic GBS, CIDP, or their variants remains unknown. Determining their prevalence is clinically relevant because wider access to serum cell-based assays for these antibodies may enable earlier diagnosis and support a shift in first-line treatment strategies.

### Guillain-Barré syndrome (GBS) and CIDP mimics

The clinical prototype of anti-pan-NF neuropathies is a “Guillain-Barré mimic” syndrome characterized by a symmetrical onset and rapid progression to respiratory failure, often with early cranial nerve involvement and dysautonomia. This presentation is frequently associated with locked-in syndrome. 9 Neuropathic pain is also an early feature and may affect up to 50% of patients. 9

Although there are isolated case reports, 10 findings typically attributed to anti-NF155 antibodies, such as upper limb tremor and ataxia, are not commonly observed in anti-pan-neurofascinopathies. 4 Additional clinical signs, such as papilledema, may also be present and should serve as red flags in patients with rapidly progressive neuropathy. 9

Nerve conduction studies typically reveal a demyelinating pattern, with significantly reduced conduction velocities, prolonged distal latencies, and increased F-wave latencies. As these disorders affect the nodo/paranodal regions, conduction blocks are characteristic, while temporal dispersion is less frequently observed. 9 Cerebrospinal fluid analysis usually shows albuminocytologic dissociation; however, as with classic GBS, normal results may be seen in early evaluations. 9

Initial diagnosis is most often GBS, but due to its potential for recurrence, many cases are later reclassified as CIDP. 4 11 Recurrent severe episodes, particularly those involving cranial nerves and respiratory failure, are highly atypical for CIDP 11 and should raise suspicion for anti-pan-neurofascinopathies. We do not know the prevalence of NF155 IgG4 that may mimic acute and monophasic phenotypes.

The IgG subclass and its complement-activating capability appears to significantly influence the clinical phenotype. Case series have described patients with IgG1 and 3 positivity who tend to present with fulminant disease courses. 4 9 The predominant subclass can shift over the course of illness. Transition from IgG1 and G3 to IgG4 has been associated with a more indolent phenotype and resistance to standard treatments, such as corticosteroids and IVIG. 4

### Combined central and peripheral demyelination (CCPD) syndrome

As a heterogeneous group of inflammatory disorders, CCPD is characterized by concomitant or rapidly sequential demyelination of the central and peripheral nervous systems, typically with a preceding infectious episode. 12 Peripheral nerve dysfunction is usually demyelinating and frequently involves the nerve roots, whereas central nervous system (CNS) involvement is variable, with patients presenting with either MS-like lesions or features resembling neuromyelitis optica spectrum disorder (NMOSD). 12 13

The etiologies of CCPD are diverse, with both hereditary and inflammatory causes included in the differential diagnosis. Among the hereditary forms, disorders such as Refsum disease, Charcot-Marie-Tooth disease associated with *GJB1* mutations, metachromatic leukodystrophy, and various mitochondrial disorders may be implicated—even presenting acutely in some cases, mimicking acquired inflammatory diseases. 14 Inflammatory causes are classically represented by anti-AQP4, anti-MOG, anti-GQ1b antibodies, paraneoplastic syndromes, and idiopathic forms. 13 14 The presence of anti-NF155 antibodies has also been associated with CCPD, given that NF155 is expressed in both the CNS and peripheral nervous system. 15 These patients typically exhibit demyelinating lesions consistent with multiple sclerosis, often demonstrating temporal and spatial dissemination, in addition to peripheral demyelination that may be clinically and neurophysiologically indistinguishable from chronic inflammatory demyelinating polyneuropathy (CIDP). 13

To date, there have been no reports of anti-pan-NF antibodies associated with CCPD. This absence may be partially explained by the fact that patients with pan-NF antibodies often present with prominent peripheral nerve involvement, potentially overshadowing CNS findings. Therefore, CCPD remains a developing clinical entity, and the possibility of anti-pan-NF involvement cannot be excluded in patients presenting with rapidly progressive, treatment-refractory demyelinating neuropathy accompanied by CNS demyelinating lesions.

### Pan-neurofascinopathies: a disease confined to the nervous system?

Pan-neurofascinopathies are conditions classically associated with the nervous system. However, a case series reported the occurrence of nephrotic syndrome in 38% of patients with IgG1 pan-neurofascinopathy, with the presence of NF186 in the kidney being a possible contributing factor. 9 16

A case presenting with papilledema, pleural effusion, thrombocytosis, and polycythemia—resembling polyneuropathy, organomegaly, endocrinopathy, myeloma protein, and skin changes (POEMS) syndrome—has been described. This patient exhibited a chronically evolving demyelinating neuropathy with temporal dispersion (uncommon in POEMS syndrome), normal VEGF levels (the principal marker of the disease), and concomitant Western blot positivity for both NF155 and 140. 17 Although the patient also had a lytic bone lesion with a localized monoclonal component, the absence of elevated VEGF and the presence of anti-NF antibodies render the case atypical, raising the possibility that, at least in part, the phenotype may be influenced by the presence of these antibodies. 17

## INSIGHTS INTO TREATMENT AND FUTURE PERSPECTIVES

Pan-neurofascinopathies are diseases typically refractory to therapies used for GBS, which is often the initial diagnosis. 4 Early administration of IVIG may provide some benefit in the disease course; however, this effect is usually transient. 4 9 Based on a case series, plasmapheresis and anti-CD20 therapies, such as rituximab, have shown potential benefits and are increasingly recommended as the cornerstone of treatment for pan-neurofascinopathies. 4

As stated previously, the prevalence of pan-NF mimicking GBS is unknown, but diagnostic indicators such as high scores on clinical scales (Egris, Erasmus, and modified Hughes), early cranial nerve involvement, ventilatory failure, and severe dysautonomia in patients over 65-years-old may guide us to prefer plasmapheresis to immunoglobulin in this phenotype due to the clinical plausibility of it being a pan-NF mimicking GBS and its variations.

In patients with severe and potentially life-threatening clinical courses, case reports have described full clinical recovery following combination therapies, such as rituximab with bortezomib or rituximab with daratumumab. 18 19

The heterogeneity in clinical response to these treatments is likely due to the dynamic nature of IgG subclass switching and its varying potential to activate the complement system. 20 Therefore, understanding the disease's immunopathological stage may be essential for selecting the most appropriate therapy. In settings where pan-NF antibody testing and subclass identification are available, early testing is recommended for all patients presenting with a fulminant GBS-like syndrome. 4

For patients with IgG1 or 3 subclasses, IVIG may be used as an initial bridging therapy, followed by early initiation of B cell depletion. 4 In contrast, for IgG4-positive patients, plasma exchange may be preferred as the initial intervention, also followed by B cell depletion. 4 In cases that are refractory to these strategies or exhibit life-threatening progression, the addition of therapies such as anti-CD38 antibodies or bortezomib may be considered. 4 18 19

Complement blockade therapies, such as ravulizumab or eculizumab, have not been used in pan-neurofascinopathies to date. However, in patients confirmed to have IgG1/3-mediated disease, these agents may serve as potential adjuncts.

For patients with IgG4 pan-neurofascinopathies who are refractory to standard therapies and have life-threatening disease, treatments with enhanced B cell depletion potential—such as CD19-directed CAR-T cell therapy—may be considered. 21 This is a rapidly acting strategy that has shown promise in other antibody-mediated diseases, such as systemic lupus erythematosus and myasthenia gravis. 21 Nevertheless, since most cases of pan-neurofascinopathy follow a monophasic course, 4 9 long-term complications like persistent hypogammaglobulinemia and recurrent infections may arise. Consequently, therapies with shorter half-lives and potential for allogeneic administration—such as CD19 CAR-NK cells—may offer more suitable immunotherapeutic strategies for monophasic autoimmune diseases. 22

Sequential monitoring of anti-NF antibody titers are advised, with the goal of achieving seronegativity following treatment. 4 Measurement of serum neurofilament light chain can also be employed during follow-up, with treatment escalation recommended in cases with rising levels. 4

Despite the severity and prolonged hospitalizations often associated with pan-neurofascinopathies, significant clinical improvement was achieved in 90% of patients in one case series, although some degree of dysfunction may persist due to axonal loss. 23 Therefore, early and aggressive initiation of treatment is essential for favorable long-term outcomes.

## CONCLUSION

Pan-neurofascinopathies are rare but potentially treatable disorders. Proper identification of red flags in patients presenting with GBS or CIDP-mimic syndromes, including its variants, along with an understanding of the immunopathological dynamics of IgG subclass switching throughout the disease course, enables the initiation of appropriate therapy. Despite the condition's severity, frequent need for ventilatory support, and prolonged hospitalizations, early and intensive treatment has the potential to yield favorable long-term outcomes. Although clinical trials supporting specific therapeutic strategies are lacking, plasmapheresis and B cell depletion (preferably to the use of IVIG) appear to be the cornerstones of management in these patients.
